# Mechanically operated signalling scaffolds

**DOI:** 10.1042/BST20221194

**Published:** 2024-04-04

**Authors:** Neil J. Ball, Samuel F. H. Barnett, Benjamin T. Goult

**Affiliations:** 1 Department of Biochemistry, Cell and Systems Biology, Institute of Systems, Molecular and Integrative Biology, University of Liverpool, Crown Street, Liverpool L69 7ZB, U.K.; 2 Max Planck Institute for Medical Research, Heidelberg 69120, Germany

**Keywords:** enzymology, epidermal growth factor receptor, mechanotransduction, molecular scaffolds, signalling, talin

## Abstract

Cellular signalling is a complex process and involves cascades of enzymes that, in response to a specific signal, give rise to exact cellular responses. Signalling scaffold proteins organise components of these signalling pathways in space and time to co-ordinate signalling outputs. In this review we introduce a new class of mechanically operated signalling scaffolds that are built into the cytoskeletal architecture of the cell. These proteins contain force-dependent binary switch domains that integrate chemical and mechanical signals to introduce quantised positional changes to ligands and persistent alterations in cytoskeletal architecture providing mechanomemory capabilities. We focus on the concept of spatial organisation, and how the cell organises signalling molecules at the plasma membrane in response to specific signals to create order and distinct signalling outputs. The dynamic positioning of molecules using binary switches adds an additional layer of complexity to the idea of scaffolding. The switches can spatiotemporally organise enzymes and substrates dynamically, with the introduction of ∼50 nm quantised steps in distance between them as the switch patterns change. Together these different types of signalling scaffolds and the proteins engaging them, provide a way for an ordering of molecules that extends beyond current views of the cell.

## Introduction

### Signalling scaffolds — controlling the flow of cellular information

With the complexity of a cell and all the moving parts, it is quite mesmerising to consider how signals from outside are received in such a way that they initiate precise signalling responses that are specific and exact within each cell. One way the cell achieves this remarkable specificity is by the precise (re)ordering of the molecules in that signalling pathway. The duration and strength of each signalling process is tightly regulated, and a lot of what we know about these pathways has come from mutations that lead to constitutive activation or deactivation, or excessive downstream signalling, as they are common drivers of diseases such as cancer.

Signalling scaffolds are non-enzymatic proteins, or regions of proteins, that bring together components of signalling pathways in time and space to co-ordinate the strength and duration of signalling cascades. Excellent reviews on signalling scaffolds already exist [1–5]; our aim here is to introduce a new class of mechanically operated signalling scaffolds. These scaffolds contain force-dependent binary switches and are built into the cell's cytoskeletal architecture and operated by actomyosin contractility.

**Figure 1.**
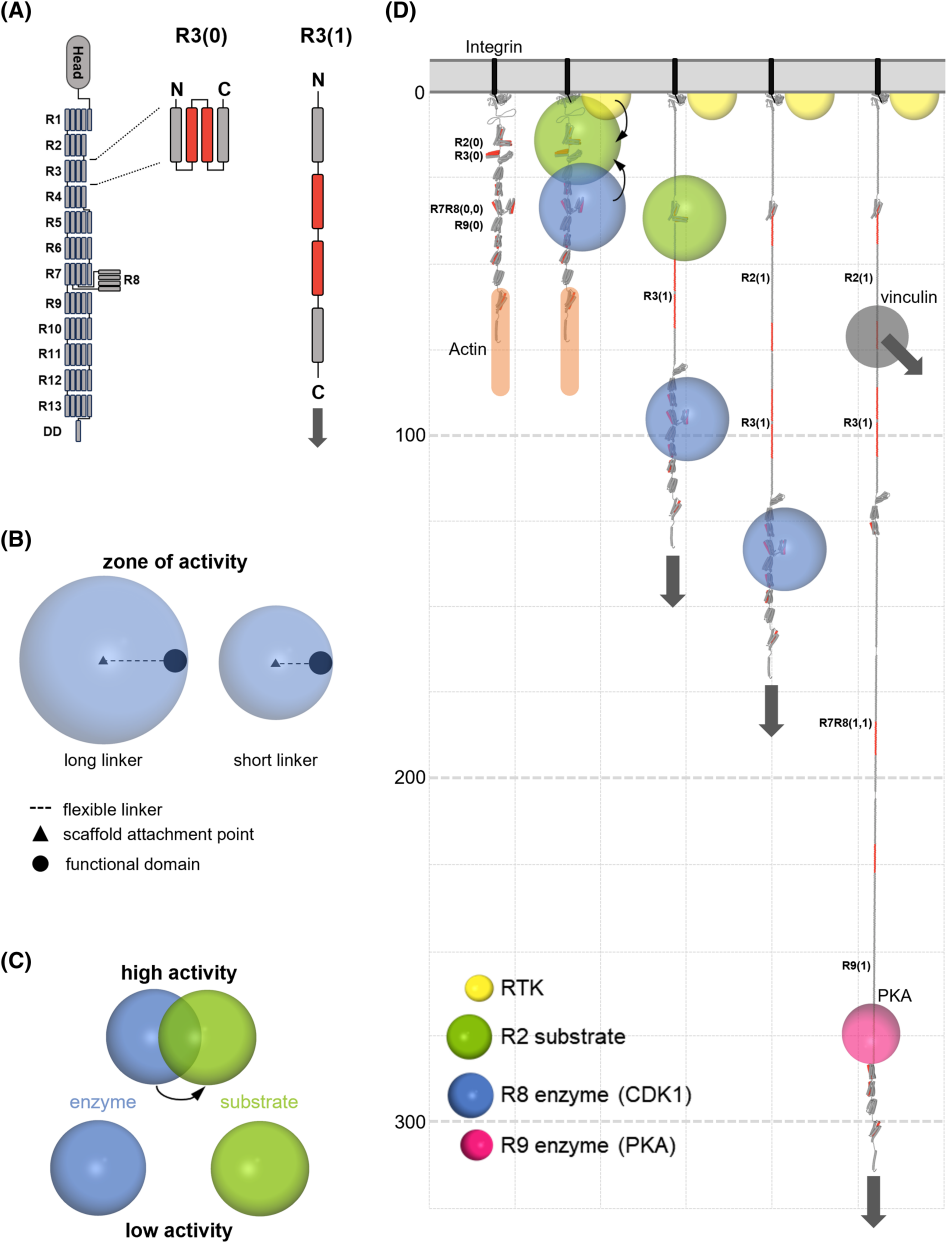
Mechanically operated signalling scaffolds. (**A**) Mechanical binary switches. Talin contains 13 force-dependent binary switches, R1–R13. The 4-helix bundle, R3, is shown as a canonical switch domain. Force converts each switch from folded ‘0’ to unfolded ‘1’ state, switching the binding partners bound to the switch and introducing a 40–50 nm extension to the scaffold downstream of this point. These states are shown as R3(0) and R3(1) to indicate the state of the specific switch. (**B**) Zones of activity. The unstructured region between a tether point and an enzymatic domain defines a zone of activity in which that enzyme has localised activity. (**C**) Mechanical control of enzymatic activity. By moving an enzyme's zone of activity relative to its substrate, the activity of the enzymatic process can be dynamically regulated. (**D**) Mechanically operated signalling scaffolds drawn to scale. Talin is an example of a mechanical linkage, connecting the ECM-integrin complexes to the actin cytoskeleton. The talin signalling scaffold contains 13 switches enabling patterns of binary information to be written into the shape of the molecule, with discrete signalling complexes assembled and spatially organised over 800 nm of space from the membrane. A five-helix switch is 5 nm in length in the 0 state but extends to ∼50 nm in the 1 state. The connections to the force generating machinery are shown by grey arrows which point in the direction of the force vector. A receptor tyrosine kinase is shown (yellow) and a substrate (green) bound to R2. CDK1 (blue) is shown bound to R8(0) and PKA (pink) is shown bound to R9(1). As the switch patterns change the location of zones of activity of enzymes bound to talin change, altering the signalling complexes. Vinculin (grey) binds to the vinculin binding sites (VBS) in talin (red) which are exposed upon domain unfolding, mediating additional links to the cytoskeleton. Only one vinculin is shown but there are 11 VBS exposed in fully extended talin. The scale on the *z*-axis (down the page) is in nanometres and only four switches are shown opening.

The idea for this mini-review arose from our analysis of what the protein talin looks like when drawn ‘to scale’ [6]. Talin is a mechanosensitive scaffold protein that couples the extracellular matrix (ECM) receptors, the integrins, to the cells’ contractile machinery, the cytoskeleton. Talin comprises a string of 13 force-dependent binary switches that can switch between folded ‘0’ and unfolded ‘1’ states in response to mechanical signals [7,8] (Figure 1A). The strings of binary switches in talin led to the realisation that information can be written into the shape of these molecules using transient changes in mechanical force. This idea of information storage in mechanosensitive proteins led to the MeshCODE theory [6,8,9] and the concept of mechanical memory. When drawn to scale it became obvious that talin represented a new class of signalling scaffold protein, one that is mechanically operated. Therefore, we chose to write this perspective article on signalling scaffolds, aiming to illustrate some of the mechanisms by which cells organise signalling pathways. One aim of this perspective is to highlight how the textbook abstract representations of these signalling complexes fail to capture their scale and as a result the potential for localised dynamic alterations in enzymatic activity are hard to see.

### Modular building blocks

The modular building blocks of signalling pathways have been worked out over the last 40 years [10–14] and include signals, receptors, enzymes, post-translational modifications (PTMs), modular binding domains that recognise PTMs, and a myriad of clever ways in which cells exploit these Lego-like blocks to string them into specific signalling pathways. To start this discussion, we introduce three additional components of these systems.

#### Mechanical linkages

The cell's cytoskeleton is incredibly complex but is built of relatively modular building blocks comprised of actin, microtubule and intermediate filaments [15]. Each part of the cell is connected via mechanical linkages to the other parts of the cell [16,17] and each cell makes mechanical linkages to its immediate surroundings, including the ECM and neighbouring cells. Many of these linkages emanate from the integrin-mediated adhesion complexes that the cell makes with the ECM. Conceptually, the force-generating machinery can be considered as being connected via mechanical linkages comprised of force-sensitive complexes to these attachment sites.

#### Mechanical binary switches

Mechanical forces in the cell arise from many sources: external changes, retrograde actin flow, or engaging the cell's motor proteins and triggering actomyosin contractility. Many proteins are mechanically regulated and a common mechanism that encodes this mechanosensitivity is the binary switch [8,18,19]. These binary switches are protein domains that have a folded ‘0’ state that can be converted to an unfolded ‘1’ state by a transient increase in tension (Figure 1A). These switches recruit different signalling molecules depending on their folding state. Strikingly the force that is required to open a switch is much higher than the force at which that switch will reset, a property called mechanical hysteresis [7,9]. Mechanical hysteresis is important in this discussion of signal transduction as it provides persistence in the switch patterns that form. In brief, each talin switch has a force threshold above which it will unfold, ranging from 5 to 25 pN force [7]. This means that a transient increase in force is required to unfold a domain. However, once unfolded a domain will only refold when the tension drops below ∼4 pN [7]. As a result, under basal cellular tension and whilst maintained in a mechanical linkage the two states of each switch domain are both stable. The integration of switches into the cytoskeletal scaffolding of the cell means they are located in precise cellular positions and held in place under tension.

#### Zones of activity

The final thing to introduce is the concept of ‘Zones of Activity’, which we define as the region of the cell where a signalling protein can operate (Figure 1B) [6]. In the case of proteins that interact with talin, the talin-binding site that tethers the protein to the scaffold is often separated from the rest of the molecule by a large, disordered linker region, meaning that each tethered protein has spatially restricted activity (Figure 1B–D). As we will illustrate, complex interdependencies can arise by moving these zones of activity relative to each other.

## Mechanically operated signalling scaffolds

Talin represents the paradigm of a mechanically operated signalling scaffold with its 13 mechanochemical binary switch domains (Figure 1A,D) serving as a mechanosensitive signalling hub [20]. One mechanism of mechanosignalling is where the location and activity of talin-binding proteins are regulated by the status of the binary switch. For example, the R8 domain binds to cyclin dependent kinase 1 (CDK1) when folded [21] but unfolding displaces this kinase and reveals a binding site for the cross-linking protein vinculin [22]. The mechanical status of the cell will determine how much CDK1 is present at adhesion sites, and differential force gradients across the cell can co-ordinate sub-cellular localisation. Similarly, the R9 domain in the open ‘1’ state exposes a mechanically-gated binding site for protein kinase A (PKA) [23] (Figure 1D). As the talin interactome continues to grow, most of the switch domains have been found to bind different ligands as a function of each switches status (reviewed in [8]). Different switch domains have different mechanical thresholds, therefore specific switch patterns are generated in response to mechanical signals. Spatially relocating signalling molecules can have multiple outcomes, it can either (i) activate signalling molecules at that site to exert a function, or (ii) sequester signalling molecules at that site, away from where they exert their function.

N.B. For conceptual purposes we only consider the binary switch in this discussion but acknowledge that other forms of mechanosensitive protein also serve as signalling scaffolds. For example, we note that the integrin tail itself which the talin engages can be considered a signalling scaffold. Similarly, p130Cas [24,25] contains a large unstructured region that gets extended in response to low forces to relocate molecules, but such a mechanism lacks the quantised states of a switch.

### Mechanical switches move enzymes and substrates relative to each other providing a Venn diagram like (re)organisation of signals and enzymatic activity

Talin, as with many mechanosensitive proteins, is tethered at both ends as it forms part of the mechanical linkages at the foundations of the cytoskeleton. This tethering means it is stretched in a single dimension away from the attachment site and along the force vector. Thus, proteins that are attached to the string of switch domains will be spatially organised by the force across the mechanical scaffold and the resultant switch pattern (Figure 1B,D).

By considering the zones of activity of the different proteins interacting with the switches, and the distance between these proteins encoded by the switch patterns, it becomes evident that another form of signalling control is occurring, where enzymes/substrates are held at specific distances relative to each other, either enhancing or suppressing enzyme activity (Figure 1C,D). Holding an enzyme close to its substrate will greatly enhance the local concentration and thus enzyme kinetics compared with when freely diffusing. The reciprocal scenario is also possible, where physically holding them apart will reduce the activity to much less than freely diffusing. Considerable changes in signalling strengths and outputs can be achieved via scaffolds spatially organising the relative positioning of molecules.

Having kinases and phosphatases in close proximity ensures an interdependence that balances the opposing signals in a way that makes the reaction metastable. A tilt in this balance, either via a change in activity of one of the enzymes, or the movement of either/both relative to the substrate(s) will cause a temporal increase in signal that persists as long as the tilted state is present. Following removal of the input signal, the balance is restored, and the local interdependence will re-establish homeostasis. However, altered switch patterns can lead to more persistent alterations to the cell's cytoskeletal architectures and repositioning of signalling molecules adorning these linkages.

### PTM of switches stabilise binary patterns, enzyme positioning and cytoskeletal architectures

PTMs affect protein structure and function, activating or inhibiting them by relieving or strengthening autoinhibition [26]. PTMs also impact on the binary switches in talin. CDK1 phosphorylation of talin weakens the R7R8 switches, making them easier to open [21]. A common feature of phosphoproteomic datasets from a variety of tissues [27,28] is that buried serine and threonine residues in the cores of talin switch domains are found to be phosphorylated. If a switch is opened to the 1 state and a previously buried residue is phosphorylated that PTM will prevent refolding, meaning the binary pattern is stabilised with that domain fixed open. As a result, chemical and mechanical signals converge to give precise conformations of the mechanosensitive scaffold proteins with the resultant cytoskeletal architectures emanating from that site maintaining the spatial organisation of the recruited signalling systems.

## Non-mechanically operated signalling scaffolds

In this section we briefly introduce the established signalling scaffolds. There are hundreds of different receptors on the surface of cells that respond to a myriad of different extracellular signals to instruct cell behaviour. Many of these, when stimulated, co-ordinate signalling scaffolds that regulate signalling pathways. Here we describe these as non-mechanically operated but it is likely that these unstructured regions are in part mechanosensitive as they can get pulled taut by actin retrograde flow or if bound proteins connect to the cytoskeleton.

### The cytoplasmic tails of receptor tyrosine kinases as signalling scaffolds

**Figure 2.**
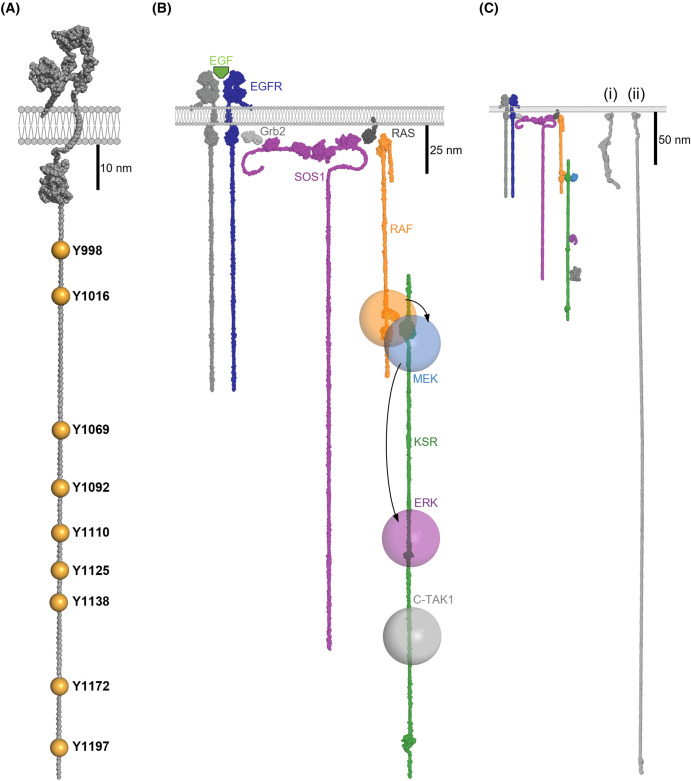
Non-mechanically operated signalling scaffolds. (**A**) The tails of growth factor receptors. The cytoplasmic tail of EGFR is shown as an example of a cytoplasmic tail serving as a signalling scaffold. The locations of the phosphorylated tyrosine residues are shown. (**B**) EGF signalling leads to EGFR dimerisation and activation of the kinase domains, which results in the tyrosine residues in the tails being phosphorylated. These phosphorylated tyrosines, in the context of their adjacent residues, create binding sites for numerous ligands. The modular adapter protein GRB2 is shown with its SH2 domain binding to the EGFR tail and the SH3 domain recruiting the guanine exchange factor, SOS1 to the membrane. SOS1 activates the small GTPase, Ras which activates the MAP kinase pathway via recruiting the kinase Raf to the PM. The kinase suppressor of Ras (KSR) is shown as an example of a cytoplasmic signalling scaffold. KSR spatially co-ordinates the locations of kinases in the MAP kinase pathway to control the flow of cellular information. KSR binds to Raf and scaffolds the MAP kinase cascade; Raf (MAPKKK), MEK (MAPKK), ERK (MAPK) positioning enzymes and substrates relative to each other on the bottom of the cell. (**C**) The EGFR and KSR signalling scaffolds and the talin mechanical signalling scaffolds drawn to the same scale. Two states of talin are shown, (i) the fully folded 13 × 0 state, and (ii) the state where 12 domains are opened. N.B. The tails are shown fully extended so as to visualise the maximum dimensions, but unless under tension they will not be stretched out like this.

Many cell surface receptors have intrinsic enzymatic activity where the activity state is controlled via external signals binding the receptor. Receptor tyrosine kinases (RTKs) represent a major class of these receptors. For example, when epidermal growth factor (EGF) stimulates the cell, it triggers dimerisation of the EGF receptor (EGFR) which activates the kinase domains. One of the major targets phosphorylated by the active kinase domains is the cytoplasmic tail of the EGFR itself (Figure 2). In terms of dimensions, the EGFR cytoplasmic tail represents a major part of the receptor (Figure 2A). The contour length of an unstructured polypeptide is ∼0.38 nm/residue [29] so these ∼220 amino acid tails can maximally span ∼84 nm, shown drawn to scale in Figure 2C. Once phosphorylated, these tails present a multitude of binding sites for SH2 (Src Homology 2) and phosphotyrosine binding (PTB) domain-containing proteins, and many proteins get relocated to the plasma membrane (PM) by binding to the active EGFR, assembling a signalling complex at the site of stimulation for the duration of the signal.

In this way the large unstructured cytoplasmic RTK tail acts as a major class of signalling scaffold, where the tail is constitutively located at the membrane, but the binding sites on the tail are only present when EGFR is stimulated. Each of the 58 known RTKs in the human kinome respond to different inputs, recruit different molecules, and trigger different outputs.

### Scaffolds that are recruited to the membrane in response to signals

One major signalling axis activated downstream of RTKs is the mitogen activated protein kinase (MAPK) pathway [30–32]. Activation of many RTKs leads to recruitment of the modular adapter GRB2 which serves to recruit the Guanine Exchange Factor, SOS1 (son of sevenless) to the PM [33]. This relocation brings SOS1 into proximity of the membrane tethered small GTPase Ras, which it activates [34,35]. Ras is a major transducer of growth signals in the cell and activation of Ras drives the recruitment of many proteins that contain Ras binding domains (RBDs) to the PM. Proteins that were previously inactive in the cytoplasm are recruited to the PM. One RBD-containing protein is the kinase Raf, and recruitment of Raf to active Ras leads to activation of Raf and the activation of the MAPK pathway (reviewed in [30,31]). The MAPK pathway contains a textbook example of a class of signalling scaffolds, the protein kinase suppressor of Ras (KSR) [36]. Initially autoinhibited in the cytosol, KSR is recruited to the Ras/Raf platform and upon engagement, unfurls to reveal its scaffold and binding sites, including those for the downstream kinases MEK and ERK, as well as other kinases such as C-TAK1 [37]. We note that this description is conceptualised and omits key interactions, for example the role of 14-3-3 proteins [38], but the point we want to make here is how the signalling scaffold, once activated, organises kinases and substrates in close proximity to each other but with defined spatial locations dictated by the zone of activity of where Ras is active and the spacing between binding sites on KSR (Figure 2B). In this way, GF binding to the receptor outside the cell leads to the precise arrangement of signalling cascades (Figure 2).

### Anchoring proteins — localised PKA signalling via A-kinase anchoring proteins

Like the examples above, A-kinase anchoring proteins (AKAPs) [23,39,40] provide another type of scaffolding in that they specifically localise PKA to sites where it can locally phosphorylate substrates. These AKAP proteins typically present a single helical motif that binds to the regulatory subunit of PKA. Many enzymes like PKA and CDKs are quite diffuse in the cell, but their activities are co-ordinated by targeted localisation at specific sites via anchoring proteins, where they are brought into proximity of substrates and other enzymes. Interestingly, as shown in Figure 1D, talin contains both PKA and CDK anchoring sites in its switch domains; with a cryptic AKAP in R9 that is revealed when R9 unfolds [23] and a CDK binding site on the surface of the folded R8 [21]. In this way, mechanical signalling scaffolds can incorporate anchoring protein functionality to precisely localise enzymes to specific locations in the cell. It is likely that many signalling scaffolds contain similar anchoring sites for other enzymes such that enzymes get specifically targeted to where they are required.

## Summary — mechanically operated signalling pathways

Great complexity can be achieved by controlling the positioning of components of signalling pathways within the cell. Scaffold proteins bring together and organise enzymes and their substrates, in a way that massively enhances their effective local concentration relative to when freely diffusing. Freely diffusing in the cytoplasm, these enzymes are dilute and the chance of a productive interaction with substrates is limited. However, once recruited to the PM their diffusion is limited to a 2D plane and the effective concentration is greatly enhanced. This role of the membrane as a signalling platform is beautifully discussed in the recent perspective by Leonard et al. [4].

The inclusion of mechanical switches into the signalling machine that is the cell, connected via cytoskeletal linkages to other regions of the cell [16], indicates an additional way for coordinating cellular responses to signals which could lead to synchronisation of the whole cell. A signal entering the cell updates the whole cytoskeletal organisation and the interconnected binary switches distributed throughout the cell will be updated with this new information. Mechanotransduction, how mechanical signals are transmitted to the nucleus, will be updated as part of this process, with explicit instructions entering the nucleus. This spatial (re)organisation of molecules means that every cell will interpret signalling pathways differently as a function of the pre-programmed, or current state of, binary patterns of information. These patterns can be persistent as the mechanical linkages are under basal tension which, combined with PTMs and proteins that limit domain refolding [41], means that these switch patterns can provide enduring alterations in the cell's cytoskeleton and mechanical computation.

Just by considering the discrete signalling scaffold systems presented here, and how the cellular organisation would look in response to different patterns of inputs, it is possible to imagine that the entire structure of the cell might be incredibly ordered. Spatial organisation of enzymes, substrates and upstream and downstream signals defines a complex network of interdependencies that both ensures robustness in linking inputs and outputs, but also provides numerous avenues for other factors to feed into these signalling pathways.

Furthermore, another layer of regulation is also in play, whereby the adorned proteins themselves are activated upon binding and/or relocalisation [42], and thus their activity is controlled by the engagement to the scaffold, which adds another layer of control and interdependence. As a result, a combination of kinetic and allosteric effects can precisely control each enzyme reaction, and this can be dynamically modulated via alterations in spatial positioning. In this way the cellular localisation of proteins and their activation states is tightly controlled and the lifetimes of each interaction will impact the duration of the signalling complex.

### Hypothesis: the synapse as a mechanically perfect cell signalling device

**Figure 3.**
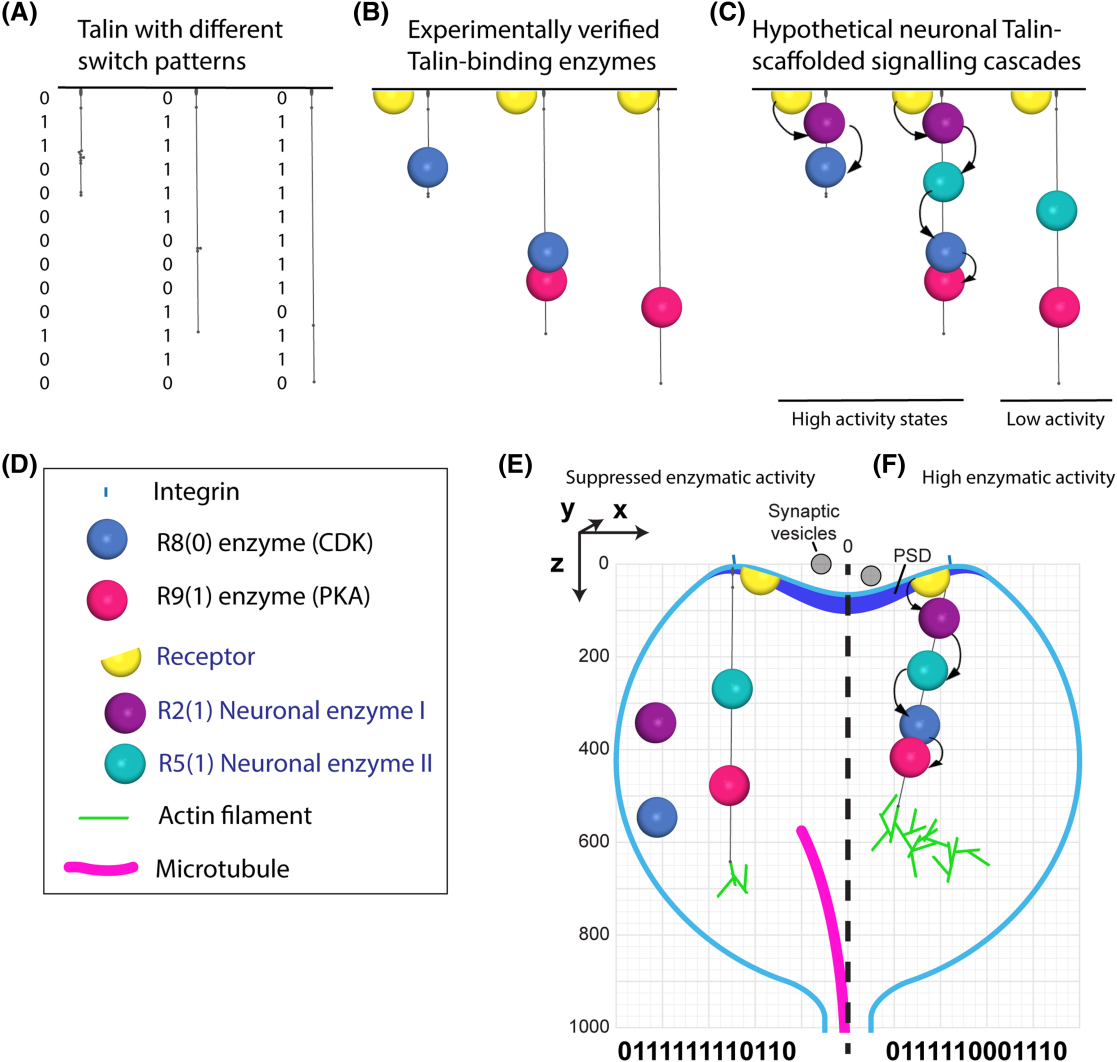
Cartoon of the spatial organisation of enzymatic cascades by talin in the synapse. All panels in this figure are drawn to the same scale to show the relative sizes of the mechanically regulated signalling scaffold protein talin, and a dendritic spine. (**A–C**) Three talin molecules are shown with different binary switch patterns. (**A**) The three talin molecules alone. (**B**) The known talin-binding enzymes, CDK1 and PKA are shown engaging the scaffold. As the switch patterns change these enzymes are spatially organised along the scaffold. (**C**) A more hypothetical example where, two as of yet unidentified neuronal enzymes that bind R2(1) and R5(1) are also included. In the left two states, efficient enzymatic activity can be envisaged as the enzymes are optimally positioned. In the right state, the enzymatic efficiency would be lower because components of the enzymatic cascade are not recruited. (**D**) Key showing the enzymes and the integrin, actin filament and microtubule to scale. (**E,F**) The hypothetical neuronal talin-scaffolded signalling cascades in (**C**) drawn in a dendritic spine. (**E**) A talin code that results in suppressed enzymatic activity. (**F**) A talin code that results in high enzymatic activity by optimising the positioning of the enzymes.

As a final reflection, we would like to present our vision of the role of signalling scaffolds in coordinating the activity of the most sophisticated cell signalling machinery there is, the brain. In particular, how mechanically-regulated scaffolding of enzymatic processes within the confines of the synapse would provide a way to dynamically regulate synaptic activity. The MeshCODE theory of a mechanical basis of memory [6,9], predicts that each synaptic junction represents a mechanically isolated cell signalling connection where electrochemical signalling leads to alterations in the neuronal cytoskeletal contractility that updates the binary switch patterns in the synaptic scaffold molecules of both the sending and receiving neuron. As discussed in this perspective and in [6,8,9] these changes in the binary switches would lead to the precise spatial (re)organisation of enzymes within the confines of the synapse. Signalling inputs would update these switch patterns, setting the spatial positioning of the enzymes coupled to the scaffolds, controlling the activity of the synapse by altering the efficiency of the enzymatic processes (Figure 3). Some codes would arrange the enzymes such that they have high activity, and some arrangements would have low activity. In this way the interplay between signalling scaffolds and signals would dynamically control the strength of each synapse's activity as a function of the information written into the switches. Perturbations of the mechanical control of signalling cascades in the brain, for example as a result of ECM stiffening [43,44], damage, or loss of mechanical homeostasis [45], might be a driver of the diseased cellular signalling observed in neurological disorders such as Alzheimer's Disease, Parkinson's Disease and in brain cancer. We propose that this organisation of signalling pathways and cytoskeletal architectures provides the basis for the brain to perform mechanical computation, and that the mechanisms described herein provide a framework for a mechanical basis of memory.

## Perspectives

- Signalling scaffold proteins are essential components of cell signalling that co-ordinate the spatial and temporal organisation of cellular signalling. We introduce a new category of mechanically operated signalling scaffolds, focussing on the protein talin as exemplar of this class.
- Signalling scaffolds are well established as hubs for controlling the flow of cellular information. However, incorporation of mechanical signalling into these scaffolds provides a way to couple mechanical and chemical signalling to dynamically regulate cell behaviour.
- The visualisation of the localised changes in enzymatic activity in response to altered mechanical cues will require visualising the shapes of molecules and their spatial positioning relative to each other. Manipulating the mechanical signals or altering the switch patterns using mutation should enable the mechanical component of signalling to be elucidated. Future research should investigate this cellular (re)organisation.

## Open Access

Open access for this article was enabled by the participation of University of Liverpool in an all-inclusive *Read & Publish* agreement with Portland Press and the Biochemical Society under a transformative agreement with JISC.

## Abbreviations

AKAP: A-kinase anchoring protein
CDK1: cyclin dependent kinase 1
ECM: extracellular matrix
EGF: epidermal growth factor
EGFR: epidermal growth factor receptor
KSR: kinase suppressor of Ras
MAPK: mitogen activated protein kinase
PKA: protein kinase A
PM: plasma membrane
PTM: post-translational modification
RBD: Ras binding domain
RTK: receptor tyrosine kinase
VBS: vinculin binding sites
